# Accumulation of toxic Pb(II) ions by the iron-containing minerals in the presence of ionic polyacrylamide soil conditioner

**DOI:** 10.1007/s11356-023-25502-x

**Published:** 2023-01-24

**Authors:** Kacper Latusek, Teresa Urban, Justyna Ulatowska, Izabela Polowczyk, Piotr Nowicki, Małgorzata Wiśniewska

**Affiliations:** 1 Department of Radiochemistry and Environmental Chemistry, Institute of Chemical Sciences, Faculty of Chemistry, Maria Curie-Skłodowska University in Lublin, M. Curie-Skłodowska Sq. 3, 20-031 Lublin, Poland; 2 Department of Process Engineering and Technology of Polymers and Carbon Materials, Wroclaw University of Science and Technology, Wybrzeże Wyspiańskiego Street 27, 50-370 Wrocław, Lower Silesia Poland; 3 Department of Applied Chemistry, Faculty of Chemistry, Adam Mickiewicz University in Poznań, Uniwersytetu Poznańskiego 8, 61-614 Poznań, Poland

**Keywords:** Lead(II) accumulation, Anionic polyacrylamide soil conditioner, Polymer-metal complex, Iron-based minerals, Aqueous suspension stability

## Abstract

The aim of this research was to determine the adsorption–desorption, surface, electrokinetic, and stability properties of aqueous suspensions of iron-containing minerals in the presence of anionic polyacrylamide (AN PAM) and lead(II) ions. Three minerals found in the soil environment, akaganeite, goethite, and magnetite, were synthesized based on the precipitation method. The interaction mechanism of heavy metal ions with polymer flocculant, which are adsorbed on the soil mineral particles, was proposed. It was shown that the best affinity to the AN PAM or/and Pb(II), adsorbed both from single and mixed solution, shows akageneite (characterized by the highly developed specific surface area). Polymer-metal complexes formed in the mixed adsorbate systems are rather stable, evidence of which is reduced desorption and consequently limited bioavailability of toxic lead ions for organisms and plants in soil environment.

## Introduction

The main sources of environmental emissions of elements such as lead are natural processes (rock weathering and volcanic eruptions) and human activity (industry, road transport, energy). Heavy metals can also be found in minerals, such as magnetite in igneous rocks as well as galena and goethite in sedimentary rocks. The spread of heavy metals in the environment occurs through a complex chain of factors (Sauve and MCBride 1998; Vanek et al. 2005; Cerqueira et al. 2011). These metals, together with the dust, end up in the air, from which they fall onto the ground or the surface waters. Plants take them up with water, and then they are absorbed by animals. The final recipient of these metals is human, who receive the greatest amount of accumulated heavy elements. Children and sick people are particularly exposed to them. Their negative impact is based on the possibility of accumulation in a living organism and chronic toxicity (Wani et al. 2015). Therefore, it is necessary to periodically control the amount of heavy metals and toxic substances in soil, air, and water, as well as to develop more and more effective methods of their separation from the natural environment (Abdel Maksoud et al. 2021, 2022, 2023).

Polyacrylamide (PAM) is a polymer used as an additive to irrigation water for farmland. It is extremely important in agriculture as it almost completely inhibits soil erosion caused by irrigation of arable land. The negative effect of PAM on crops has not been documented (Guzzo and Guezennec 2015). The addition of polyacrylamide increases the cohesion of the substrate and preserves the roughness of the surface. Soil particles in the soil–water phase bind better with each other, so that soil erosion does not occur (Ben-Hur and Keren 1997; Green et al. 2004). The action of polyacrylamide is beneficial not only from the point of view of agrotechnical operations but also has an important ecological aspect, because the greater amounts of pesticides, fertilizers, heavy metals, and other harmful substances are retained in the cultivated soil and does not move in the environment (Wiśniewska et al. 2015; He et al. 2019; Fijałkowska et al. 2020a, b; 2021).

Iron oxides and hydroxides are a very important group of minerals, both from the economic (iron ore) and soil science (oxidation–reduction processes, soil-forming processes) points of view (Waychunas et al. 2005). The most important representatives of this group are akaganeite, goethite, magnetite, hematite, limonite, and lepidocrocite. Akaganeite is iron oxyhydroxide (β-FeOOH). It can be found in natural environment that is strongly acidic and contains high levels of chloride, such as geothermal brines and sea-floor nodules; it is also formed as a corrosion product of some meteorites (Xiong et al. 2008; Zhao et al. 2012). It is also an important constituent in various kinds of soils. Goethite is iron(III) oxyhydroxide (α-FeO(OH)) with 62.8% of this element. It is found in gas bubbles in volcanic rocks in the oxidation zone. It can accumulate in sedimentary formations, creating ore deposits of marine, lake, or marsh origin. In soils, goethite is one of the main coloring substances, in the form of submicroscopic grains, evenly distributed throughout the soil mass (Giménez et al. 2007; Zhoua et al. 2010; Mokwenye et al. 2016). Magnetite is tri-iron tetroxide (Fe_3_O_4_) with an iron content of 72.4%. It is a common side mineral of many igneous rocks, such as gabbro and basalt, and is present in many sedimentary and metamorphic rocks. In soils, magnetite comes from parent deposits and takes the form of single, irregular grains. It is present in almost all soils, but its content is usually very low (Grimley and Arruda 2007; Ahmed and Maher 2018; Luo et al. 2018). These iron-containing minerals have high sorption capacities for metal and anionic contaminants such as arsenic, chromium, lead, mercury, and selenium.

Taking into account the above facts, the main aim of this study was to determine the adsorption–desorption, surface, electrokinetic, and stability properties of aqueous suspensions of iron-containing minerals in the presence of anionic polyacrylamide and lead(II) ions. Three minerals occurring in soil environment were selected, namely, akageneite, goethite, and magnetite. They were obtained by synthesis using chemical reagents based on the precipitation method. The most probable structure of mixed adsorption layers formed at the mineral-aqueous solution interface and the binding mechanisms of individual adsorbates were proposed. This is crucial for understanding the interaction of heavy metals with polyacrylamide flocculants that are adsorbed on the soil mineral particles surface. The literature reports characterize insufficiently the simultaneous adsorption of polymeric substances and heavy metal ions in the systems containing iron oxides and hydroxides. The previous studies proved the effectiveness of these synthetic minerals in adsorption removal of As(III) from aqueous solutions (Polowczyk et al. 2018; Ulatowska 2022).

## Experimental

### Synthesis and characterization of iron-containing minerals

To obtain the akageneite, 8.11 g of FeCl_3_ was dissolved in 500 cm^3^ of deionized water (the initial pH of the solution amounted to 1.1). To the solution, 0.1 M NaOH was slowly added until the pH of the mixture reached about 10. During NaOH addition, the mixture was stirred using a magnetic stirrer. After getting the required pH, the mixture was stirred for 1 h. The precipitate formed was separated from the solution by centrifugation. The separated residue was then washed with demineralized water until neutralization is reached (pH about 7.0) and dried in air at room temperature (298 K).

In order to synthesize goethite, 1 M NaOH was dropped into a mixture of 0.5 M FeSO_4_ and 0.1 M Fe_2_(SO_4_)_3_ in a 1000 cm^3^ beaker. The solution was stirred with a magnetic stirrer during the addition of NaOH. NaOH was added until the pH of the mixture reached about 7–8. After getting the required pH, the mixture was stirred for 1 h. Next the precipitate was separated by centrifugation and washed with demineralized water (until pH about 7.0 was obtained) and dried in air at room temperature (298 K).

Magnetite preparation started with dissolution of 6.1-g FeCl_3_∙6H_2_O and 4.2-g Fe_2_SO_4_∙7H_2_O in 100 cm^3^ of deionized water. Then the obtained solution was heated to 90 °C. Once the desired temperature was reached, a mechanical stirring of the solution began, and 10 cm^3^ of ammonium hydroxide (25%) was added. The stirring was continued for another 30 min, maintaining the abovementioned temperature. After this time, the mixture was cooled to room temperature. The black magnetite precipitate formed during the reaction was with demineralized water until neutralized (pH about 7.0) and then maximally separated from it using neodymium magnet disks. The resulting wet deposit was dried at 313 K.

After drying, all synthetic products were gently crushed and ground with a mortar to obtain a powder (to facilitate its dosage).

The specific surface area of synthetic iron-containing adsorbents (akaganeite, goethite, and magnetite) was measured by the Brunauer–Emmett–Teller (BET) method for the helium/nitrogen mixture by using a FlowSorb 2300 apparatus (Micromeritics Instruments Corp, Norcross, GA, USA). The particle size distribution of the investigated materials was determined using an LS13320XR Particle Size Analyzer (Beckman Coulter, Brea, CA, USA). The density of synthetic materials was determined using a pycnometer. The morphological images were taken using an Axio Imager.M1m optical microscope (Zeiss, Jena, Germany) and a JSM-6610LV scanning electron microscope (JEOL Ltd., Akishima, Japan) after sputtering the samples with carbon using an automatic coating machine JEC-530 (JEOL Ltd., Akishima, Japan). The XRF technique (Epsilon 5 spectrometer, PANalytical, Malvern, UK) was used to obtain the elemental composition of minerals. Moreover XRD analysis (Empyrean diffractometer, PANalytical, Malvern, UK) enabled confirmation of their crystal structure.

### Characterization of adsorbates

The adsorbate used in the research was anionic polyacrylamide (AN PAM) delivered by Korona and lead(II) nitrate (source of Pb(II) cations) produced by Sigma-Aldrich. The weight average molecular weight of the polyacrylamide was 14,000,000 Da. This polymer contains 30% of carboxyl groups, which are the source of negative charge of its macromolecules. The pK_a_ value of AN PAM obtained by the potentiometric titration method (Minczewski and Marczenko 1985) was 3.2. Based on the determined pK_a_ point, the degree of AN PAM ionization at a given pH was calculated using the Henderson-Hasselbalch equation (Minczewski and Marczenko 1985). The degree of dissociation changes from 38.7 (at pH 3), through 98.4 (at pH 5) and 99.98 (at pH 7), to 99.99% (at pH 9).

The NaCl solution with a concentration of 0.01 mol/dm^3^ was used as the supporting electrolyte. All measurements were carried out at 25 °C. The adsorption–desorption and stability measurements were performed at pH 5, which is typically noted for commonly occurring acidic soils.

### Spectrophotometric studies

Spectrophotometry was applied to determination of both adsorbed and desorbed amounts of AN PAM and Pb(II) ions as well as stability of synthetic minerals suspensions.

Before the main adsorption experiments, the calibration curves were prepared (linear dependencies of absorbance versus the adsorbate concentration). The adsorption measurements were made by the static method with three polymer concentrations (10, 50, and 100 ppm) at pH 5 using 0.01 g of akaganeite, 0.08 g of goethite, and 0.03 g of magnetite (for 10 cm^3^ of the solution). The examined samples were shaken for 24 h using OLS 200 water bath (Grant). During this time, the pH was controlled and adjusted as needed. The reaction of AN PAM with a hyamine proposed by Crummet and Hummel (Crummett and Hummel 1963) was applied. The solution absorbance (coming from white color of the solution) was measured after 15 min using the UV–VIS spectrophotometer (Carry 1000; Varian) at 500 nm. To determine the concentration of Pb(II) ions, the PAR method was used (Dagnall et al. 1965). This method is based on Pb(II) reaction with 4-(2-pyridylazo)-resorcinol (PAR) in an ammonium buffer which results in red-colored PAR-Pb chelate complex. The corresponding absorbance was determined at 520 nm. The desorption possibilities of both adsorbates were defined using NaOH solution with concentration of 0.01 mol/dm^3^.

In order to determine the stability of the examined suspensions without and with AN PAM or/and Pb(II) ions, the changes in their absorbance as a function of time were measured. A total of 20 cm^3^ of NaCl with a concentration of 0.01 mol/dm^3^ was introduced into the beaker, and then the previously weighed mineral with a mass of 0.03 g was added. The suspension was sonicated for 3 min, and the pH was adjusted to value 5.0 ± 0.1. The suspension prepared in this way was shaken in a thermostatic water bath for 30 min; meanwhile its pH was controlled. In the next stage, the suspension was transferred into a quartz cuvette and subjected to spectrophotometric measurement for 10 h (40 measurements every 15 min). Next, 20 cm^3^ of the solution was prepared by adding the appropriate amount of the polymer or/and lead(II) ions as well as supporting electrolyte, so that the final concentration of the adsorbates was 10 ppm and the concentration of the NaCl solution was 0.01 mol/dm^3^. In total, 0.03 g of the mineral was added to the prepared solution, and its stability was measured.

### Potentiometric titrations and electrokinetic studies

Studies were performed in the following systems: mineral (akaganeite, goethite, magnetite) + NaCl; mineral (akageneite, goethite, magnetite) + NaCl + AN PAM; mineral (akageneite, goethite, magnetite) + NaCl + Pb(II); and mineral (akageneite, goethite, magnetite) + NaCl + AN PAM + Pb(II). Due to precipitation of lead hydroxide at pH above 6, all measurements in the presence of lead(II) ions were carried out in the pH range 2–6.

The potentiometric titrations (Janusz 1999) of iron-containing minerals with and without AN PAM or/and Pb(II) (with concentrations 10 ppm) were performed in the thermostated Teflon vessel (0.085 g of akaganeite or 0.1 g of goethite or magnetite was added to 50 cm^3^ of appropriate solution). The suspensions were titrated using NaOH solution with concentration 0.1 mol/dm^3^ in the pH range 3–11. The measuring set consisted of thermostat RE 204 (Lauda), electrodes: calomel and glass (Beckman Instruments), pH meter PHM 240 (Radiometer), laboratory stirrers, and automatic microburette (765 Dosimat, Metrohm). The solid surface charge density was calculated with the special program Titr_v3 (author W. Janusz).

The iron-containing mineral samples (without and with AN PAM or/and Pb(II) ions (with concentrations 10 ppm)) for the electrophoretic mobility measurements were prepared adding 0.03 g of the solid to 200 cm^3^ of the examined solution. The obtained suspension was divided into 8 equal portions and poured into flasks. In each flask, the appropriate pH value 2, 3, 4, 5, 6, 7, 8, and 9 (± 0.1) was adjusted. The electrophoretic mobility was measured using the Zetasizer Nano ZS with the universal dip cell (Malvern Instruments). The special computer program was applied for the calculation of zeta potential of solid particles based on the Henry equation (Oshima 1994). For all examined suspensions, the mean aggregate sizes were also measured at pH 5 applying Zetasizer Nano ZS.

## Results and discussion

### Characterization of iron-containing minerals

**Fig. 1:**
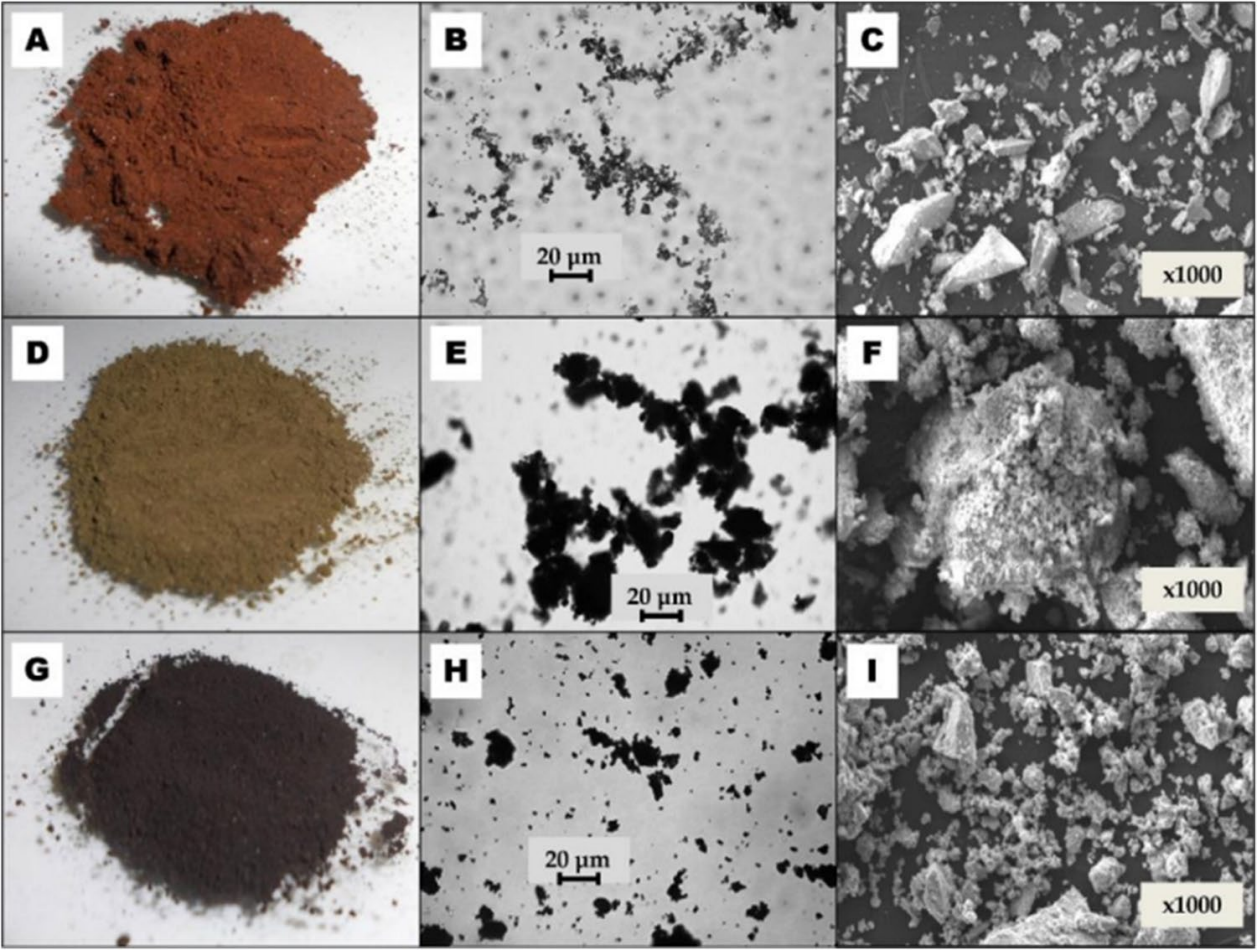
Images of synthetic iron-based minerals taken with a digital camera (**A**, **D**, **G**), an optical microscope (**B**, **E**, **H**) and a scanning electron microscope (**C**, **F**, **I**); viewed from above: akaganeite (**A–C**), goethite (**D–F**) and magnetite (**G–I**)

The iron-containing minerals have standard coloration — akaganeite is brownish-red, goethite is yellowish-green, and magnetite is black (Fig. 1). In addition, it was observed that magnetite is the only one with magnetic properties that help separate the adsorbent from the solution. The particle size of iron-containing minerals is evident in optical microscope images. Particles several hundred nanometers in size are visible next to aggregates of about a few tens of microns. Akaganeite has both fine particles and larger particles (Fig. 1A). In contrast, the size and shape of goethite and magnetite are similar (Fig. 1E and H). The characteristic parameters of the particle size distribution are included in Table 1 along with the basic textural parameters of the obtained iron-based minerals (Schwermann and Cornell 2000).

**Table 1:** Physicochemical characteristics of iron-containing minerals

Mineral	Specific surface area [m^2^ g^−1^]	Particle-size distribution [μm]	Span	Density [g cm^−3^]
Akaganeite	236.5	d_10_ 2.27; d_50_ 18.8; d_90_ 95.4	4.95	3.45
Goethite	13.75	d_10_ 5.02; d_50_ 20.3; d_90_ 188.4	36.5	4.37
Magnetite	36.94	d_10_ 5.69; d_50_ 50.8; d_90_ 223.8	4.29	5.35

**Fig. 2:**
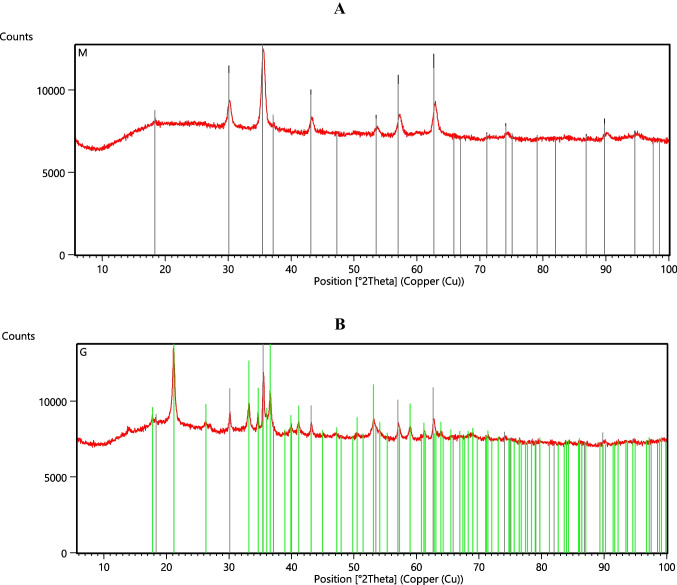
XRD spectra of goethite (**A**) and magnetite (**B**)

Analysis of the data in Table 1 indicate that akaganeite has the most developed specific surface area, whereas goethite — the least developed specific surface area. The determined particle size distribution analysis shows that the produced iron-containing adsorbents are characterized by a wide particle size distribution (span > 4). The elemental composition of examined minerals was presented in Table 2. It was shown that Fe content for akaganeite is the lowest (50. 724 wt%), whereas for magnetite — the highest (70.881 wt%). The XRD spectra confirmed the crystal structure of goethite and magnetite (Fig. 2), whereas akageneite sample is amorphous.

**Table 2:** Elemental composition of iron-containing minerals

Element	Akaganeite	Goethite	Magnetite
	Concentration [wt%]		
Si	1.493	-	0.434
P	0.191	0.141	0.151
Cl	0.026	-	0.02
K	0.003	0.001	0.018
Ca	0.147	0.074	0.091
V	0.107	-	0.098
Cr	0.003	-	0.131
Fe*	**50.724**	**62.42**	**70.881**
Ni	0.010	-	0.019
Cu	0.005	0.011	-
Zn	0.007	-	0.005
Zr	0.003	-	-
Ag	0.045	-	-
W	0.056	0.061	0.057
S	-	1.27	0.871
Mn	-	0.041	0.056
As	-	0.001	-

*Fe is main element of obtained minerals

### Adsorption–desorption properties of iron-containing minerals toward anionic polyacrylamide or/and lead(II)

**Fig. 3:**
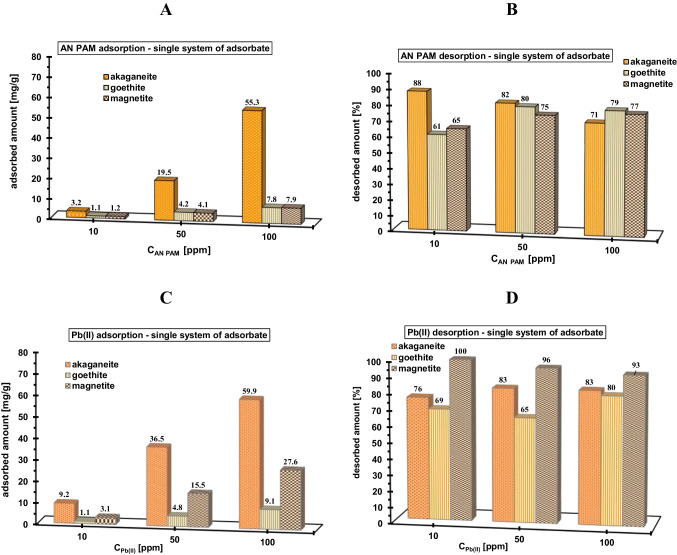
Adsorbed and desorbed amounts of AN PAM (**A**, **B**) and Pb(II) ions (**C**, **D**) in the single adsorbate systems containing the examined minerals

**Fig. 4:**
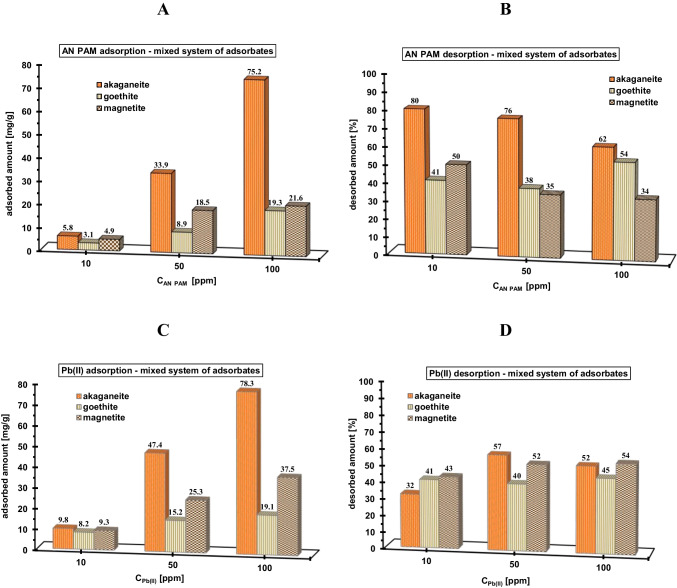
Adsorbed and desorbed amounts of AN PAM (**A**, **B**) and Pb(II) ions (**C**, **D**) in the mixed adsorbates systems containing the examined minerals

**Fig. 5:**
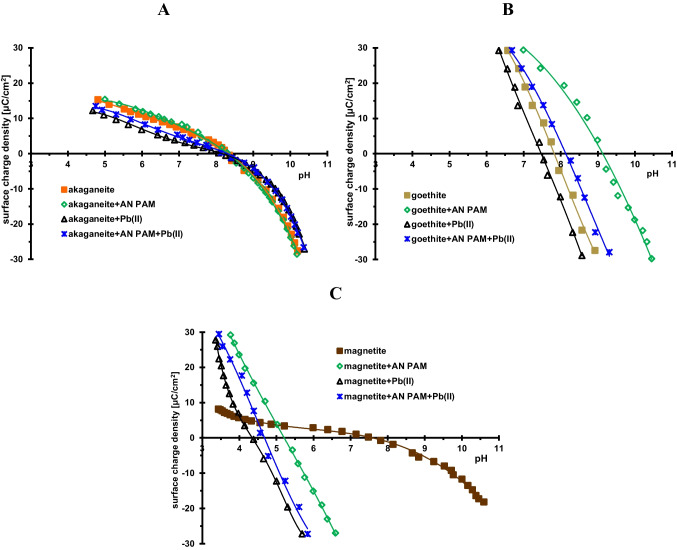
Surface charge density of the examined minerals without and with AN PAM or/and Pb(II) ions for **A** akageneite, **B** goethite, and **C** magnetite

The adsorbed and desorbed amounts of AN PAM and Pb(II) ions from the systems containing single and mixed adsorbates and examined minerals are presented in Figs. 3 and 4. These measurements were performed at pH 5, at which polymeric chains are totally dissociated and solid surfaces are positively charged (Fig. 5). The points of zero charge (pzc) of the examined solids have the following values: approx. 8.4 for akaganeite, 7.8 for goethite, and 7.6 for magnetite. In the pH range below pH_pzc_, the surface of the mineral is positively charged, which favors the adsorption of anionic polymer, whereas in the pH range of above pH_pzc_ — with a negative charge, which in turn is manifested by electrostatic attraction with Pb(II) cations. The polymer and heavy metal adsorption increases with initial concentration of both AN PAM and Pb(II) ions, which is rather obvious behavior. The greatest adsorption level of both adsorbates from the single system is observed on the akaganeite surface (for AN PAM, it reaches value 55.3 mg/g and for Pb(II) — 59.9 mg/g) (Fig. 3A and C). Such behavior is a result of the greatest specific surface area of akaganeite (236.5 m^2^/g) in comparison to the other two iron-containing solids (Table 1). Besides electrostatic attraction between anionic polymer and positively charged surface of examined adsorbents, the hydrogen bonds can be formed (through AN PAM carboxylic groups and mineral hydroxyl groups) (Kasprzyk-Hordern 2004). The similar adsorption affinity of applied minerals was observed in relation to Pb(II) cations — the greatest adsorbed amounts of heavy metal ions occur on the akaganeite surface (Fig. 1C). Despite electrostatic repulsion at pH 5 between lead cations and positive solid surface, the chemical bonds can be formed in such adsorption system (Jiang et al. 2010), leading to the effective binding of the toxic element. The desorption of both adsorbates from their single solutions using NaOH with concentration 0.01 mol/dm^3^ is rather effective in all examined system, and it changes in the range 61–100%.

In the mixed system of adsorbates, where both components were added simultaneously, the increase of both AN PAM and Pb(II) ion adsorption takes place (Fig. 4A and C). The main reason of such situation was creation of polymer-metal complexes, which is facilitated by the electrostatic attraction between oppositely charged substances (Gu et al. 2018). These complexes can have inter- and intramolecular structure, due to the divalent character of lead cations. Their formation causes noticeable increase of adsorbed amounts of heavy metal ions, which bind to multiple dissociated carboxyl groups within one or two polymeric chains, thereby increasing the level of adsorption of anionic polyacrylamide (for AN PAM, adsorbed amount reaches value 75.2 mg/g and for Pb(II) — 78.3 mg/g) (Fijałkowska et al. 2020a, b). Such mechanism can lead to the formation of adsorption multilayers. The desorbed amounts of both adsorbates decrease for all examined systems (Figs. 4B and 3D). The desorption changes in the range 32–80%, which may indicate a stronger binding of both adsorbates in a complexed form, which reduces their bioavailability for soil organisms and plants (Fijałkowska et al. 2021).

### Surface and electrokinetic properties of iron-containing minerals in the presence of anionic polyacrylamide or/and lead(II)

The surface properties of minerals were characterized on the basis of the dependence of the surface charge density as a function of solution pH determined by the potentiometric titration method. The results of these measurements are presented in Fig. 5. As was mentioned above the pzc values of the examined solids are approx. 8.4 for akaganeite, 7.8 for goethite, and 7.6 for magnetite. The presence of lead(II) ions usually reduces the surface charge of the mineral. It is associated with the formation of additional negatively charged surface groups, which in turn is forced by their interaction with heavy metal cations (Skwarek et al. 2008). On the other hand, the addition of anionic PAM generally causes a noticeable increase in the surface charge density as a result of the interaction of AN PAM negative carboxyl groups with the surface groups of the mineral and the formation of their negatively charged variant (Chibowski and Wiśniewska 2001). Systems containing both adsorbates show an intermediate behavior. The surface charge density dependencies are located between the corresponding curves relating to systems containing single adsorbates. Thus, the abovementioned effects of creating of an additional number of adsorption sites with opposite charges overlap, resulting in the obtained values of the solid surface charge density.

**Fig. 6:**
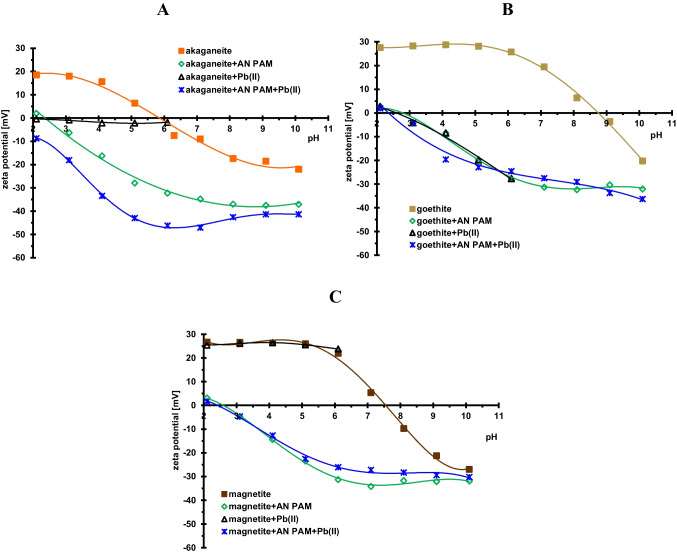
Zeta potential of the examined minerals without and with AN PAM or/and Pb(II) ions for **A** akageneite, **B** goethite, and **C** magnetite

Figure 6 shows the changes in the zeta potential of the iron-containing minerals without and in the presence of an anionic polymer or/and lead(II) ions as a function of the solution pH. This parameter describes the slipping plane area, informing about the sign and magnitude of the charge accumulated there. The isoelectric points (iep) of solids, for which the zeta potential is zero, are at the following pH values: approx. 5.1 for akaganeite, 8.8 for goethite, and 7.3 for magnetite. The presence of the adsorption layers of the macromolecular compound results in a marked reduction of the electrokinetic potential of solid particles in the whole examined pH range. The mechanism of this phenomenon is complex and results from many processes that accompany the adsorption of long polymeric chains. They include the following: (1) the shift of slipping plane from the solid surface due to the formation of polymeric layers of considerable thickness, (2) the presence of dissociated carboxyl groups of adsorbed AN PAM macromolecules in the slipping plane area (related to segments present in loop and tail structures), and (3) the change in the ionic composition of this area of the electrical double layer (edl), resulting from the desorption of the basic electrolyte ions, which is a consequence of the bonding of polyacrylamide chains (M’Pandou and Siffert 1987). The overlapping of all these effects results in a specific value of the electrokinetic potential. Similar relationships were observed in the presence of the mixed AN PAM + Pb(II) adsorption layer. In this case, adsorption of polymer-metal complexes at the solid–liquid interface causes the same effects as the adsorption of polyacrylamide itself. It results in a specific structure of the surface layer and influences the electrokinetic properties of the examined systems. The influence of Pb(II) ions on the zeta potential of solid particles in the pH range 2–6 is of different nature dependently of mineral type. The greatest changes in the electrokinetic potential are observed for goethite, whereas in the case of magnetite, practically no effect occurs.

### Stability properties of iron-containing minerals in the presence of anionic polyacrylamide or/and lead(II)

**Fig. 7:**
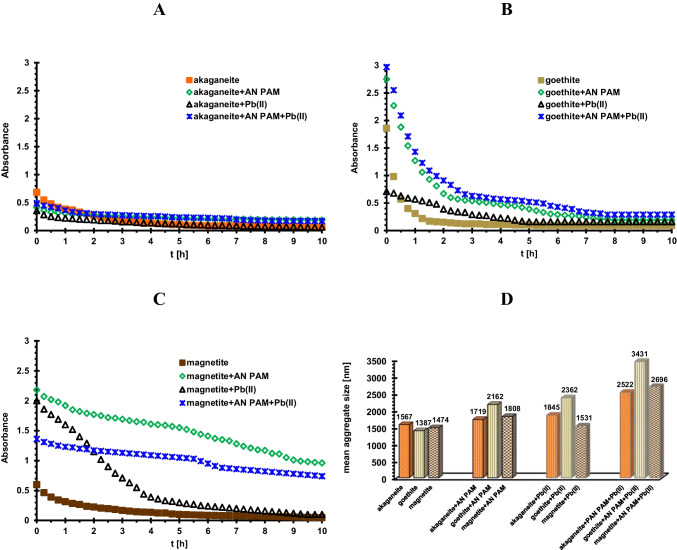
Stability of the examined mineral suspensions without and with AN PAM or/and Pb(II) ions for **A** akageneite, **B** goethite, and **C** magnetite; **D** mean size of aggregates formed in the studied systems

The electrokinetic properties of aqueous suspensions of solids can be significantly modified due to the formation of adsorption layers on the surface of their particles. This is manifested by specific changes in the stability of this type of colloidal systems, which is presented in Fig. 7A–C. They represent the changes in system absorbance over 10 h without and in the presence of AN PAM or/and Pb(II). The lower the value of this parameter, the more unstable the suspension is.

For all examined suspensions, their stability decreases with time. The systems without adsorbates are the less stable the more their pH_iep_ points are close to 5 (changes in absorbance were recorded at this pH value). It is due to the more effective coagulation of systems under conditions where the zeta potential is close to zero. Moreover, the addition of adsorbates results in a greater or lesser increase in the system stability (except for akageneite, for which the observed changes are minimal). The increase in suspension stability in the presence of anionic polyacrylamide is primarily caused by electrosteric interactions originating from adsorbed polymer layers. Repulsion between individual solid particles, or their smaller aggregates, is a result of not only the spatial obstacle, but also the electrostatic repulsion between the AN PAM adsorption layers. The effect of stability increase in systems containing only lead(II) cations is noticeably smaller than in the case of the anionic polymer. It is caused by the electrostatic stabilization mechanism that appears as a result of the adsorption of bivalent metal ions. In the case of mixed adsorbates, the binding of AN PAM-Pb(II) complexes leads to the formation of adsorption layers with a specific structure. Its presence contributes to an increase in the stability of the suspension, which is primarily the result of electrosteric interactions (Napper 1984).

Figure 7D presents the average size of mineral particle aggregates without and covered with polymer or/and heavy metal adsorption layers. For all studied minerals, the greatest changes in their size were observed in the presence of mixed adsorbate systems. Although the addition of individual adsorbates generally causes increase of the suspension stability, this does not translate into a reduction in the size of formed aggregates. This is especially noticeable in systems containing polymer-metal complexes. Their adsorption can be multilayered, and therefore aggregates composed of a small number of particles, but covered with adsorption films of considerable thickness, can be formed. Due to the loose structure of these aggregates (flocks), they do not tend to sediment but rather to remain in the bulk phase of the solution.

## Conclusions

The greatest adsorption levels of both adsorbates from the single and mixed solutions were observed on the akaganeite surface (in single systems: for AN PAM — 55.3 mg/g and for Pb(II) — 59.9 mg/g; in mixed systems: for AN PAM — 75.2 mg/g and for Pb(II) — 78.3 mg/g). Such behavior is a result of the greatest specific surface area of akaganeite (236.5 m^2^/g) in comparison to the other two iron-containing solids and formation of polymer-metal complexes in mixed solution, which adsorption can lead to multilayers creation. The desorbed amounts of both adsorbates from their single solutions using NaOH with concentration 0.01 mol/dm^3^ change in the range 61–100%, whereas from mixed solutions it is in the range 32–80%. This indicates stronger binding of both adsorbates in a complexed form, which reduces their bioavailability for soil organisms and plants.

Changes in the surface charge density of minerals caused by the presence of the polymer or/and lead(II) ions result primarily from the formation of additional surface groups with a specific charge sign, which is a consequence of the adsorption of anionic PAM or Pb(II) cations. The greatest changes in the zeta potential of the solids suspensions (compared to the system without adsorbates) occur in the presence of anionic polyacrylamide, which is caused by the specific conformation of adsorbed macromolecules affecting the structure of the electrical double layer at the interface. The slipping plane shift and changes in the ionic composition of this area are mainly responsible for modification of the electrokinetic properties of the examined suspensions in the presence of AN PAM or/and Pb(II) ions.

Stability of aqueous suspensions of mineral particles at pH 5 is rather low — the systems are the less stable the closer their pH_iep_ points are the value 5 (coagulation efficiency increases). The addition of the anionic polymer generally results in an increase in the stability of aqueous mineral suspensions due to the electrosteric interactions between the adsorbed AN PAM layers. The loose structure of aggregates of solid particles formed in a mixed system of adsorbates manifests itself in a significant increase in their size, simultaneously in maintenance a low tendency to sedimentation.

## Data Availability

Not applicable.
